# Clinical Implications of Atrial Fibrillation Detection Using Wearable Devices in Patients With Cryptogenic Stroke (CANDLE-AF) Trial: Design and Rationale

**DOI:** 10.3389/fcvm.2022.837958

**Published:** 2022-04-04

**Authors:** Sodam Jung, Hye Ah Lee, In Sook Kang, Sang Hoon Shin, Yoonkyung Chang, Dong Woo Shin, Moo-Seok Park, Young Dae Kim, Hyo Suk Nam, Ji Hoe Heo, Tae-Hoon Kim, Hee Tae Yu, Jung Myung Lee, Sung Hyuk Heo, Ho Geol Woo, Jin-Kyu Park, Seung-Young Roh, Chi Kyung Kim, Young-Soo Lee, Jin Kuk Do, Dong-Hyeok Kim, Tae-Jin Song, Junbeom Park, CANDLE-AF Trial Investigators

**Affiliations:** ^1^Division of Cardiology, Ewha Womans University Mokdong Hospital, Seoul, South Korea; ^2^Clinical Trial Center, Ewha Womans University Mokdong Hospital, Seoul, South Korea; ^3^Division of Cardiology, Ewha Womans University Seoul Hospital, Seoul, South Korea; ^4^Department of Neurology, Ewha Womans University Mokdong Hospital, Seoul, South Korea; ^5^Department of Neurology, Ewha Womans University Seoul Hospital, Seoul, South Korea; ^6^Department of Neurology, Yonsei University College of Medicine, Seoul, South Korea; ^7^Division of Cardiology, Severance Cardiovascular Hospital, Seoul, South Korea; ^8^Division of Cardiology, Kyung Hee University Hospital, Seoul, South Korea; ^9^Department of Neurology, Kyung Hee University Hospital, Seoul, South Korea; ^10^Division of Cardiology, Hanyang University Seoul Hospital, Seoul, South Korea; ^11^Devision of Cardiology, Korea University Guro Hospital, Seoul, South Korea; ^12^Department of Neurology, Korea University Guro Hospital, Seoul, South Korea; ^13^Division of Cardiology, Daegu Catholic University Medical Center, Daegu, South Korea; ^14^Department of Neurology, Daegu Catholic University Medical Center, Daegu, South Korea

**Keywords:** atrial fibrillation, wearable device, single-lead ECG, rhythm monitoring, ischemic stroke, cryptogenic stroke

## Abstract

**Background:**

Although many electrocardiography wearable devices have been released recently for the detection of atrial fibrillation (AF), there are few studies reporting prospective data for wearable devices compared to the strategy of the existing guidelines in the detection of atrial fibrillation (AF) after cryptogenic stroke. A tiny single-patch monitor is more convenient than a conventional Holter monitor recording device and, therefore, longer duration of monitoring may be acceptable.

**Methods and Design:**

The CANDLE-AF study is a multicenter, prospective, randomized controlled trial. Patients with transient ischemic attack or ischemic stroke without any history of AF will be enrolled. The superiority of the 72-h single-patch monitor to standard strategy and non-inferiority of the 72-h single-patch monitor to an event-recorder-type device will be investigated. Single-patch monitor arm will repeat monitoring at 1, 3, 6, and 12 months, event-recorder-type arm will repeat monitoring twice daily for 12 months. The enrollment goal is a total of 600 patients, and the primary outcome is the detection of AF which continues at least 30 s during study period. The secondary outcome is the rate of changes from antiplatelet to anticoagulant and major adverse cardiac and cerebrovascular events within 1 year.

**Conclusions:**

The results of CANDLE-AF will clarify the role of a single-lead patch ECG for the early detection of AF in patients with acute ischemic stroke. In addition, the secondary outcome will be analyzed to determine whether more sensitive AF detection can affect the prognosis and if further device development is meaningful. (cris.nih.go.kr KCT0005592).

## Introduction

For stroke patients, the American/European Stroke Society recommends 24–72 h rhythm monitoring for detecting atrial fibrillation (AF) as well as additional monitoring with long-term noninvasive monitors or implantable loop recorders (ILRs) if the cause of the stroke is unclear (1–4). The recently issued European Society of Cardiology 2020 AF guideline (3) recommends intensive electrocardiogram (ECG) monitoring in high-risk patients older than 75 years (Class of recommendation: IIa). ILRs can monitor the ECG rhythm 24 h a day for more than 3 years and can detect AF considerably more often than stepwise additional monitoring including 24-h Holter, which is the guideline-based standard method (12.2% vs. 2.0% and 30% vs. 3% in 12 and 36 months, respectively, after cryptogenic stroke; *n* = 221 vs. 220; *p* < 0.001) (5–7). However, because ILR insertion is an invasive procedure, not all patients receive ILR monitoring. The Early Treatment of Atrial Fibrillation for Stroke Prevention Trial (EAST-AFNET 4) (8) showed that early rhythm control of AF improved major clinical outcomes. Therefore, the development of a convenient, effective, noninvasive ECG monitor is valuable for diagnosing post-stroke AF.

Although the accuracy, sensitivity, and specificity of single-lead ECG recording have improved (9), few prospective studies have compared the AF-detection rates between different types of single-lead ECG recorders in patients with cryptogenic stroke. Studies such as the Apple Heart Study (10), REHEARSE-AF (REmote HEArt Rhythm Sampling using the AliveCor heart monitor to scrEen for Atrial Fibrillation) (11), and the SCREEN-AF (SCREENing for Atrial Fibrillation) (12) proved the usefulness of single-lead ECG, their results were based on data from the general population and not data that was specifically obtained from patients with an ischemic stroke or transient ischemic attack (TIA). In 2020, a study of nurse-led monitoring during stroke (SPOT-AF) demonstrated the feasibility and efficacy of a single-lead ECG recorder for post-stroke AF detection (13). A prospective study for comparing the event recorder (2 times daily) with a 7-day Holter monitor is ongoing (14).

According to the 2020 guideline of the European Society of Cardiology (3), single-lead ECG recording using a wearable device can be used for confirming a diagnosis of AF (Class of recommendation: Ia). A recent systematic review and meta-analysis suggested a noninvasive rhythm-monitoring strategy prior to invasive monitoring (15). Against this background, we designed a trial that compares a single-lead patch to an event-type recorder and standard care, respectively. We aimed to determine whether a single-lead patch-type ECG recorder is superior to the standard methods and to ascertain the non-inferiority of single-lead ECG patch recorder to an event recorder for early detection of AF in high-risk patients who have experienced an acute stroke and, thereby, facilitate an early switch from antiplatelet to anticoagulant medication based on the findings and other clinical conditions. Consequently, in this study, we intend to 1) reveal the clinical utility of a single-lead patch ECG for AF detection in patients who have experienced acute stroke; 2) identify whether single-patch ECG monitoring has possibility to be another widely used monitoring method for the detection of AF after cryptogenic stroke; and 3) explore, as a pilot study, the effect of the difference in the detection rate of AF on the recurrence of TIA or ischemic stroke and major adverse cardiovascular and cerebrovascular events (composite of nonfatal stroke, nonfatal myocardial infarction, and cardiovascular death) in patients with AF after stroke.

## Materials and Methods

### Trial Design

The Clinical implications of Atrial fibrillatioN Detection using a wearabLE device in patients with cryptogenic stroke (CANDLE-AF) study is a multicenter, prospective, open-label, randomized, controlled trial for detecting AF in post-stroke patients who have not been previously diagnosed with AF (reg. no. cris.nih.go.kr KCT0005592). Following the detection of AF in patients with cryptogenic stroke, we will conduct a superiority trial of single-lead patch ECG monitoring against standard monitoring and a non-inferiority trial of single-lead patch ECG monitoring against event-recorder type monitoring.

### Primary Objectives

For 12 months, this trial will investigate the superiority and non-inferiority of a 3-day continuous single-lead ECG patch in the detection of AF after stroke or TIA in comparison with the conventional strategy (guideline-based group) and event-recorder type monitoring, respectively.

### Secondary Objectives

In each group, we will evaluate the rate of change from antiplatelet therapy to anticoagulants following AF detection and the rate of major adverse cardiovascular or cerebrovascular events, which include all-cause mortality, stroke or TIA, and all-cause hospitalization and major adverse cardiovascular events (composite of nonfatal stroke, nonfatal myocardial infarction, and cardiovascular death). As the treatment policy will be changed in accordance with the detection of AF, we will assess the change in the incidence of recurrent stroke within 6 months/12 months of the initial stroke in each group.

### Study Population and Randomization

**Figure 1:**
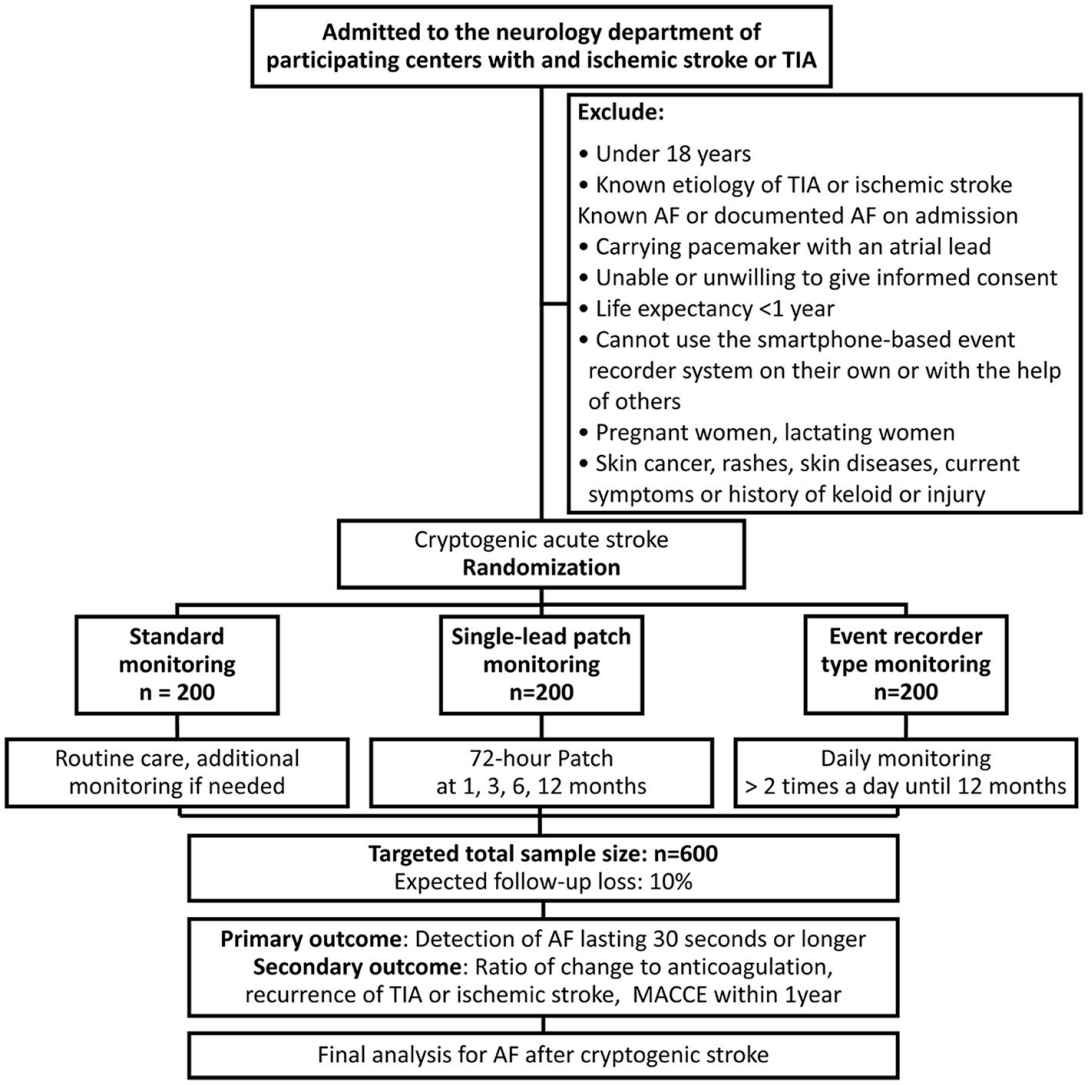
Flow chart of the study design. AF, atrial fibrillation; TIA, transient ischemic attack; MACCE, major adverse cardiovascular and cerebrovascular events (composite of nonfatal stroke, nonfatal myocardial infarction, and cardiovascular death).

Seven tertiary hospitals with stroke units in South Korea will participate in this trial and these centers represent a full coverage of all levels of care, including state-of-art tests, monitoring, imaging equipment, the latest treatment policies of specialists, and intensive care units. All patients who first visit the department of neurology with a stroke or TIA without history of AF at the time of admission and if no AF was detected during monitoring of the hospital stay will be enrolled in this study after obtaining voluntary informed consent. In this study, by referring to the inclusion criteria used in CRYSTAL-AF (16), the minimum symptoms required for inclusion by TIA were established: speech or language deficit, weakness of an arm or leg, or hemianopsia. As an inclusion criterion, history of AF was established as a person who did not have AF at the time of admission and who had no prior AF diagnosis. A surface ECG at hospitalization will be used as the screening test to check for pre-existing AF. During the hospital stay, continuous ECG monitoring will be performed through telemonitoring. Before being discharged, all enrolled participants will be randomized to the: 1) the standard treatment group, 2) the single-lead ECG patch group, and 3) the event-recorder group. The exclusion criteria are described in Figure 1. We performed block randomization using random number generator function of Excel (Microsoft, USA). The randomization ratio is 1:1:1.

### Two Types of Single-Lead ECG Monitors

**Figure 2:**
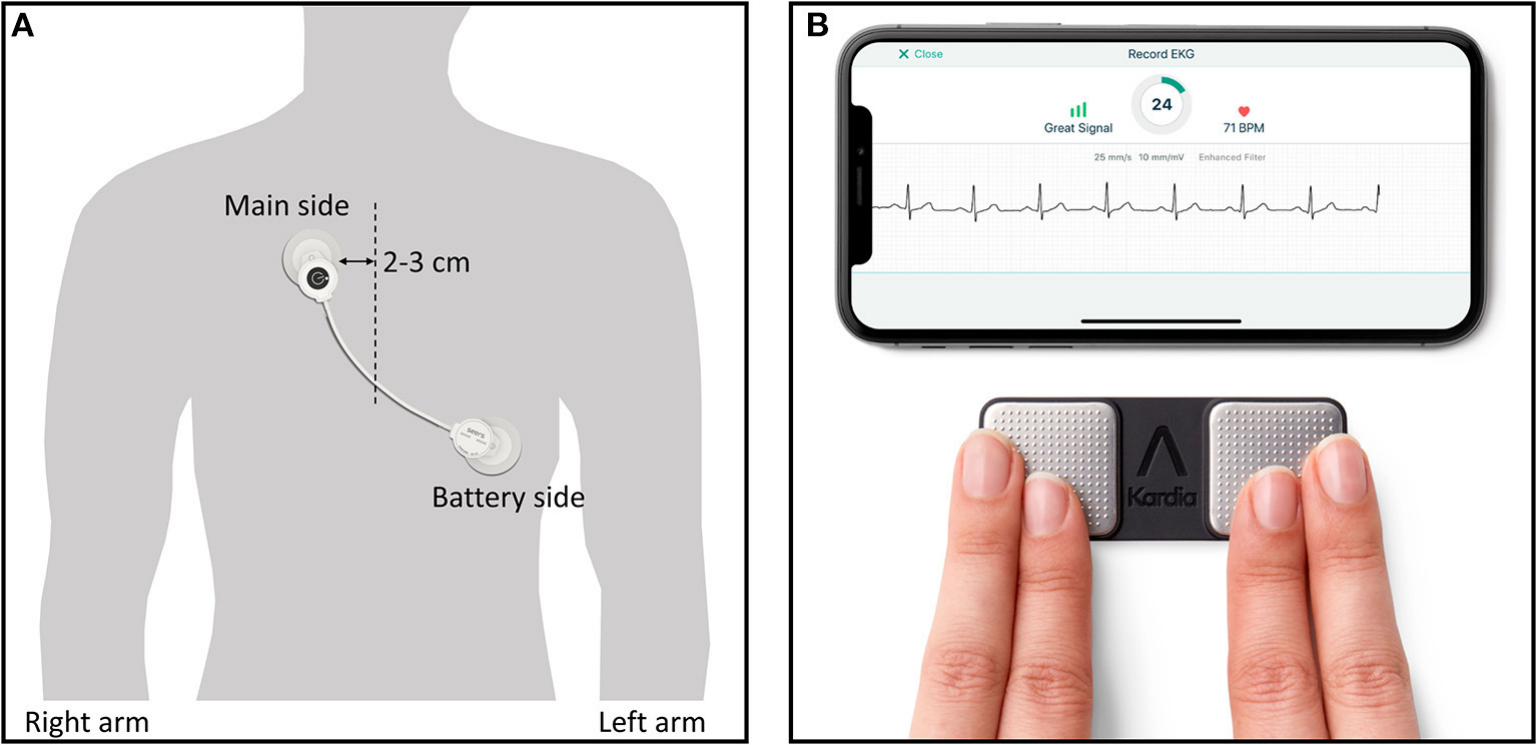
Two types of wearable devices in this study. **(A)** Single-lead ECG monitor (mobiCARE-MC100 TM) which is attached by replaceable adhesive ECG electrodes, **(A)** Single-lead event recorder type monitor (KardiaMobile systemTM) being used with finger of right and left hand touching the respective electrode and showing sample ECG rhythm in mobile phone display. Copyright with permission from Seers Technology **(A)** and Alivecor **(B)**.

A recently developed wearable device for the detection of arrhythmia, the adhesive single-lead ECG patch (mobiCARE-MC100 TM, Seers Technology, Seongnam-si, Gyeonggi-do, Republic of Korea), which comprises a light chest patch weighing 9.2 g without any other additional parts, allows long-term continuous ECG monitoring and is relatively more comfortable than standard Holter monitoring (Figure 2A). In a study comparing this single-lead patch monitor and Holter by wearing them simultaneously for 24 h, most patients did not feel discomfort with single-lead patch monitor (17). Based on these results, it is thought that the single-lead patch was more comfortable than the Holter. Monitoring is possible for up to 72 h when the patch is used once, and it is possible to continue the monitoring even when the battery has been replaced. Patients can check their ECG through a mobile phone application and the ECG will be automatically transmitted to a core laboratory. This single-lead ECG patch uses an artificial intelligence-based algorithm to systematically classify and analyze data to improve diagnostic accuracy. Furthermore, this device has an advantage in terms of signal accuracy because it has excellent ability to remove motion artifacts that may be mistaken for a heartbeat. According to a comparative study where a Holter monitor and the abovementioned single-lead ECG patch were simultaneously attached to non-arrhythmic patients, the intraclass correlation coefficients for total QRS complexes, ventricular ectopic beats, and supraventricular ectopic beats of the two devices were 0.991, 0.999, and 0.966, indicating that the performance of the two devices did not differ significantly (17).

An event recorder-type ECG device based on a smartphone (Kardia Mobile TM, Alivecor Inc., San Francisco, CA, USA) has been developed (Figure 2B) and can enable the patient to measure and transmit the heart rhythm for a specific number of times a day and has superior AF-detection ability when compared to routine care in non-AF patients (11). In our study, in addition to the superiority of the single-lead patch to standard monitoring, we intend to demonstrate the non-inferiority of the single-lead ECG patch type monitoring to the event-recorder type of monitoring.

The server to which the ECG is transmitted is managed by the manufacturer of each device, and access to the data is restricted to those authorized to handle the data related to patient care and this study by IRB approval. The device manufacturers had no role in the trial design, data accrual, or analysis.

### ECG Monitoring

All participants will receive ECG monitoring for at least 24 h in the stroke unit and a separate 12-lead ECG recording will be performed. In a simulation study using CRYSTAL-AF trial data (18), simulated intermittent monitoring data in the 12 months after a cryptogenic stroke, although AF detection doubles at 2 months, showed that a 30-day event recorder (sensitivity of 22.8%) and a quarterly repeated 7-day Holter monitor (sensitivity of 20.8%) have the highest sensitivity among various short-term or periodic monitoring strategies. In the EMBRACE (19) trial, a study using an external loop recorder for 30 days, AF was found in 42 (14.8%) of 284 patients within 4 weeks, 21 (7.4%) patients were detected within the first week. Based on this, we thought that it would be more cost-effective to monitor only once every 3 months than to monitor 2 or 3 months respectively. On the other hand, it is unclear whether AF found in monitoring after 1 year has a causative role for index stroke (15). This is because risk factors for stroke or factors that stroke patients usually have, such as metabolic disease and old age, may be the cause of AF found by longer monitoring (20). For 1 year monitoring, it is also reasonable to try quarterly monitoring for 3, 6, 9, and 12 months. However, considering the patients', 1, 3, 6, and 12 months of monitoring were planned for 6 and 12 months. Therefore, the 3-day single-lead patch group will receive for 72 h of monitoring within 1 month and at 3, 6, and 12 months. The event-recorder group will start monitoring at 7 days after the stroke and monitoring will be repeated twice daily for 12 months. For the standard treatment group, 24-h Holter monitors as a minimum will be initially performed, and the decision to perform subsequent tests is left to the physician's choice preferably according to the latest guidelines (3, 21). Study ECG data from the standard group will be analyzed by trained physicians and the ECG data from both wearable devices will be transmitted to a cardiac core laboratory for analysis. For single-patch devices, all AFs automatically detected by the software in 72-h ECG recordings are visually supervised by experts to ensure that the AF diagnosis is accurate. In case of single-patch device, all detected AF by software automatically in 72-h ECG recordings will be visually inspected by experts to ensure that they were consistent with AF episodes. The results of the core laboratory analysis, if indicated, will be communicated telephonically to the individual participant and the patient's physician as soon as possible but no later than 3 weeks after detection by the enrolling study center. The study-specific definition of clinical AF is a recording of AF lasting 30 s or longer on an ECG, as defined in the 2020 European Society of Cardiology guideline for the diagnosis and management of AF (3) (Table 1).

**Table 1:** Summarization of CANDLE-AF study protocol.

	**Enrollment**	**Hospital discharge**	**0 month**	**3 months**	**6 months**	**12 months**
Standard group			On-demand additional evaluation			
Smartphone-based monitoring group	Randomization	24-h Holter	Daily 2 times of monitoring by smartphone-based monitor			
Single-lead patch monitoring group			72-h single-lead patch #1	72-h single-lead patch #1	72-h single-lead patch #1	72-h single-lead patch #1

### Clinical Monitoring

For 12 months following hospitalization, the detection of AF recorded on the device, all-cause mortality, all-cause rehospitalization, and change to anticoagulation will be recorded at the outpatient visit or through a telephone call. The prevalence of stroke, cardiovascular disease, diabetes, hypertension, and heart failure will be ascertained using the International Classification of Diseases-10 code corresponding to the diagnosis in the medical record. Sex, age, and the results of general blood tests, biochemical tests, myocardial enzyme levels, echocardiography, and brain imaging tests performed during the hospitalization period will be recorded. It is intended to be used as a covariate when comparing differences in primary or secondary outcomes.

### Study Duration, Interim Analyses, and Early Termination

The difference in the detection rate of AF is the primary outcome. The device-based monitoring will be stopped when AF is detected. The secondary outcome is the rate of changes from antiplatelet to anticoagulant, major adverse cardiac and cerebrovascular events (composite of nonfatal stroke, nonfatal myocardial infarction, and cardiovascular death), and occurrence of major bleeding (fatal or overt bleeding with a drop in hemoglobin level of at least 2 g/dL or requiring transfusion of at least 2 units packed blood cells, or critical anatomical site hemorrhage (e.g., intracranial, retroperitoneal) within 1 year. For secondary outcome, the follow-up period is 12 months. Changes in antiplatelet and anticoagulant therapy will not affect clinical study discontinuation. Monitoring and follow-up will be terminated early if a skin disease occurs due to the patch, if the patient no longer wants to participate, or if it is impossible for the patient to participate due to causes, such as hospitalization due to a serious disease or death. When discontinuation or drop-out occurs, all data of the participants that were recorded up to that time point will be used, and patient data up to the point of interruption in the intention-to-treat strategy will be used for statistical analysis. If the participant does not want their data to be used, all of their data will be discarded and not used in the statistical analysis.

### Sample-Size Estimates

The sample-size calculation is based on the primary endpoint: “the detection probability for each group.” The detection probability was assumed as standard treatment: 2.5%, smartphone recorder: 8.5%, single-lead device: 14.5%, based on the SPOT-AF trial (13) and a previous assessment of a simulation in the CRYSTAL-AF trial (18). Non-inferiority margin was assumed to be 2.3% conservatively based on the difference in expected detection rates between single-lead device (14.5%) and smartphone-based device (8.5%) (13, 18). First, for determining the single-lead device's superiority to standard strategy at a significance level (alpha) of 5%, power (1-beta) of 80%, and margin of 2.3%, we calculated that 108 participants are needed in each group, assuming a 10% drop-out rate. 2, to prove the single-lead monitor's non-inferiority to the event recorder with a significance level (alpha) of 5%, power (1-beta) of 80%, and margin of 2.3%, each group needs 200 patients with a 10% drop-out rate. For randomization, each group will recruit 200 participants by applying the results of the pair with the greater number of participants (Figure 1). The sample-size calculation was performed using the Power and Sample Size website (http://powerandsamplesize.com/Calculators/Compare-2-Proportions/2-Sample-Non-Inferiority-or-Superiority, accessed November 22, 2021). Because it is an intention-to-treat study, we plan to conduct analyzes including drop-out cases except which the subject wants to remove the data.

### Statistical Analysis

The CANDLE-AF trial will use an intention-to-treat analysis that includes all participants according to randomization. It is hypothesized that, regarding the AF-detection rate, the 72-h single-patch monitoring will be superior to standard care and non-inferior to the event-recorder type of monitoring. For the baseline variables, bivariate relationships will be investigated using chi-square or Fisher's exact tests and Student's *t*-tests or Wilcoxon rank sum tests. All continuous variables will be represented as mean or median with standard deviation or interquartile range, respectively. And according to the results of the normality test performed by the Shapiro-Wilk test, the *t*-test is performed for data following the normal distribution, otherwise the Mann-Whitney U test will be performed. Categorical and dichotomized variables will be described as percentages and analyzed using Fisher's exact test.

The primary outcome will be compared between the 72-h monitoring group and each control arm using the chi-square test or Fisher's exact tests. Unadjusted outcome effect sizes will be estimated as odds ratios with 95% confidence intervals as appropriate. In addition, for the time to the first documented AF episode during the 12-month observation period, Kaplan–Meier curves will be calculated for each arm and compared using a log-rank test. Moreover, we will compare the total major adverse cardiovascular or cerebrovascular events and the rate of change from antiplatelet to anticoagulant therapy following AF detection, as well as the incidence of recurrent strokes. All analyses will evaluate the effectiveness through multivariate analysis, taking other factors into account in a progressive model. In multivariate analysis, age, hypertension, heart failure, valvular disease, history of myocardial infarction, thyroid insufficiency, obesity, chronic obstructive pulmonary disease, chronic kidney disease, and smoking, which are known independent risk factors for Afib (22–27), are planned to be used as covariate. The Statistical Package for the Social Sciences (SPSS version 26.0, IBM SPSS Statistics, Armonk, New York, USA) will be used for statistical analyses. *P* < 0.05 will be considered statistically significant.

### Current Status

The CANDLE-AF trial is planned to complete the 3-year enrollment period for the prespecified 600 participants from the 7 participating centers. The first participant was enrolled in November 2020, and~ 100 patients were enrolled by the end of November 2021. Enrollment may be completed in late 2023, and the primary results of the CANDLE-AF trial will be available by early or mid-2024.

### Ethical Conduct

The study protocol was approved by the Independent Ethics Committee of the Ewha Womans' University Mokdong Hospital, Seoul, Korea (EUMC 2020-08-004-004), and all participating centers obtained approval from their corresponding ethics committees. All study procedures comply with the principles of Good Clinical Practice and the Declaration of Helsinki. Only patients who have provided written informed consent based on sufficient explanation will be included.

## Discussion

Several types of ECG monitoring strategies after cryptogenic stroke have been investigated, and most studies have proved that longer monitoring has a higher AF-detection rate. Martin et al. (28) reported the results of 72-h Holter compared to 24-h Holter in cryptogenic stroke (2.50 vs. 4.30%, total *n* = 1,135). A German prospective randomized study with 7-day continuous ECG monitoring in a stroke unit (29) showed a detection rate of 7.69%, which is significantly greater than the 2.83% of 24-h Holter monitoring. In studies using 10-day Holter (FIND-AF trial; Finding Atrial Fibrillation in Stroke–Evaluation of Enhanced and Prolonged Holter Monitoring) (21) and a 30-day external loop recorder (EMBRACE trial) (19), the detection rates were 14% (*n* = 398) and 16.1% (*n* = 572), respectively. In the PER DIEM study (Post-Embolic Rhythm Detection with Implantable vs External Monitoring), 1-year ILR was better than 30-day ELR: 15.3% vs. 4.7% (RR 3.29) (30). Use of ILR for 3 years confirmed AF-detection rates of up to 41.4% (31).

However, the conventional Holter is uncomfortable and difficult to use for a long time, and ILRs can be used comfortably for a long time but are invasive. Then, there have been limitations to extensive long-term ECG monitoring. Moreover, a recently published large study showed that 7 days of monitoring was not long enough to make a significant difference compared to conventional strategies (standard vs. 7-day Holter until discharge in the stroke unit, 4.0% vs. 5.8%; total *n* = 3 465) (32).

To overcome these limitations, monitoring methods for AF have undergone technological advances, and novel devices have been developed that may improve their feasibility, comfort, and cost-effectiveness. The current spectrum of devices and methods for AF involves intermittent rhythm-monitoring strategies using blood pressure monitors and handheld devices and continuous ECG recordings of variable durations through wearable, dry-electrode belts, and adhesive patches (20, 33).

The single-lead ECG patch (mobiCARE-MC100 TM) allows continuous monitoring and transmission to the core laboratory and is relatively comfortable because of the lightweight design. An event recorder-type ECG device based on a smartphone (KardiaMobile systemTM) that can measure and transmit the ECG predetermined intervals has also been developed. Each of these monitoring tools has advantages and disadvantages; however, they are more likely to detect AF compared to the conventional strategy. In patients over 65 years of age with elevated CHADS-VASc score (≥ 2) without AF, REHEARSE-AF (REmote HEArt Rhythm Sampling using the AliveCor heart monitor to scrEen for Atrial Fibrillation) (11) reported a 3.9-fold increase (3.8%, 19/500 vs. 1.0%, 5/500) in the AF detection rate by using the smartphone-based event-recorder type system twice weekly over 12 months, compared to routine care. A trial comparing event-recorder-type ECG with the standard guidelines for post-stroke patients also showed superiority for AF detection (8.5% vs. 2.8%, total *n* = 588) (34).

This trial is conducted to prove that the single-lead patch monitoring device is superior to the methods in the existing guidelines and is non-inferior to the event-recorder-type device. As a design for efficient research performance, the interval of use of single-lead patch devices was determined by referring to the period of high detection rate revealed in the previous ILR study for patients with cryptogenic stroke (6). If AF can be detected noninvasively and conveniently but sensitively, physicians could have more chances to reduce the embolic event rates and improve the prognosis of stroke patients. In addition, with this trial, we plan to monitor the patients' long-term outcomes; therefore, we expect additional information on whether more AF findings will lead to better patient outcomes. We hope to suggest better monitoring guidelines for post-stroke patients to detect more AF cases.

Recurrent TIA or ischemic stroke in 3 years showed no significant difference in the standard group and ILR group (9% vs. 11%, total *n* = 441; *p* = 0.64) (5, 6). The Find-AF-randomized trial demonstrated no significant difference in recurrent stroke at 12 months in subclinical AF patients of 10-day Holter monitoring group and control group (3.7% in 200 patients vs. 5.4% in 198 patients; *p* = 0.46) (21). As such, there have been several attempts to elucidate the relationship between the more sensitive detection of silent AF after cryptogenic stroke and the prognosis, but there were no results showing a clear difference.

### Study Limitations

In this study, we will monitor AF after cryptogenic stroke using three types of devices. Although we will randomize the enrolled patients, this trial is an open-label trial because of the differences in the shape of the device and the format of the result sheet. Therefore, it is difficult to completely rule out detection bias in the diagnosis of AF detection. In addition, the follow-up will be carried out for only 1 year. Thus, the trial does not compare long-term outcomes according to differences in the detection rates of AF. If there is a significant difference in the AF-detection rates, further study will be needed to compare the long-term outcomes. Several single-lead patch ECG recording devices have been validated for AF detection, but single-lead patch device which we used in this study has not yet been validated for AF detection. Although this is a limitation of our study, we are trying to secure specificity by examining all detected AFs by experts. Another limitation is that the control arm is “usual standard treatment arm” without a structural unified diagnostic protocol and that may vary among doctors and thus may create a bias both in favor or against the suggested treatment strategy.

## Conclusion

More frequent and longer ECG monitoring by convenient devices after stroke has the potential to be used as a non-invasive, inexpensive, and effective way to increase AF detection, which could improve the secondary prevention of recurrent stroke.

## Ethics Statement

The studies involving human participants were reviewed and approved by Independent Ethics Committee of the Ewha Womans' University Mokdong Hospital, Seoul, Korea (EUMC 2020-08-004-004). The patients/participants provided their written informed consent to participate in this study.

## Author Contributions

JP: conceptualization. JP and SJ: methodology. SJ: writing—original draft and visualization. SJ and HL: statistical methodology. SJ, IK, D-HK, SS, YC, DW, T-JS, M-SP, YK, HN, JH, T-HK, HY, JL, SH, HW, J-KP, S-YR, CK, Y-SL, JD and JP: writing—review and editing. All authors contributed to the article and approved the submitted version and take responsibility for all aspects of the reliability and freedom from bias of the data presented and their discussed interpretation.

## Funding

This research was supported by the Basic Science Research Program funded through the National Research Foundation of Korea (NRF) by the Ministry of Science, ICT & Future Planning [Grant Number: NRF-2017R1E1A1A01078382] as well as the Korea Medical Device Development Fund grant funded by the Korean government [grant number: 9991006899, KMDF_PR_20200901_0234, NTIS, KMDF-RnD 202014X28-00 (RS-2020-KD000234)]. One hundred single-lead type recorders (cost: ~USD 10,000) were donated by the manufacturer.

## Conflict of Interest

The authors declare that the research was conducted in the absence of any commercial or financial relationships that could be construed as a potential conflict of interest.

## Publisher's Note

All claims expressed in this article are solely those of the authors and do not necessarily represent those of their affiliated organizations, or those of the publisher, the editors and the reviewers. Any product that may be evaluated in this article, or claim that may be made by its manufacturer, is not guaranteed or endorsed by the publisher.
