# Corrigendum: Clinical implications of atrial fibrillatioN detection using wearabLE devices in patients with cryptogenic stroke (CANDLE-AF) trial: Design and rationale

**DOI:** 10.3389/fcvm.2023.1199185

**Published:** 2023-04-19

**Authors:** Sodam Jung, Hye Ah Lee, In Sook Kang, Sang Hoon Shin, Yoonkyung Chang, Dong Woo Shin, Mooseok Park, Young Dae Kim, Hyo Suk Nam, Ji Hoe Heo, Tae-Hoon Kim, Hee Tae Yu, Jung Myung Lee, Sung Hyuk Heo, Ho Geol Woo, Jin-Kyu Park, Seung-Y. Ro, Chi Kyung Kim, Young-Soo Lee, Jin Kuk Do, Dong-Hyeok Kim, Tae-Jin Song, Junbeom Park

**Affiliations:** ^1^ Mokdong Hospital, Ewha Womans University School of Medicine, Seoul, South Korea; ^2^ Ewha Womans University Seoul Hospital, Seoul, South Korea; ^3^ College of Medicine, Yonsei University, Seoul, South Korea; ^4^ Severance Cardiovascular Hospital, Seoul, South Korea; ^5^ Kyung Hee University Medical Center, Seoul, South Korea; ^6^ Hanyang University Seoul Hospital, Seoul, South Korea; ^7^ Korea University Guro Hospital, Seoul, South Korea; ^8^ Daegu Catholic University Medical Center, Daegu, North Gyeongsang, South Korea

**Keywords:** atrial fibrillation, wearable device, single-lead ECG, rhythm monitoring, ischemic stroke, cryptogenic stroke

A Corrigendum on Clinical implications of atrial fibrillation detection using wearable devices in patients with cryptogenic stroke (CANDLE-AF) trial: Design and rationale By Jung S, Lee HA, Kang IS, Shin SH, Chang Y, Woo Shin D, Park M-S, Kim YD, Nam HS, Heo JH, Kim T-H, Yu HT, Lee JM, Heo SH, Woo HG, Park J-K, Roh S-Y, Kim CK, Lee Y-S, Do JK, Kim D-H, Song T-J, Park J and CANDLE-AF Trial Investigators. (2022) Front. Cardiovasc. Med. 9:837958. doi: 10.3389/fcvm.2022.837958

**Incorrect Funding**

In the published article, there was an error in the Funding statement. The funding statement for the Korea Medical Device Development Fund grant funded by the Korean government was displayed as [grant number: 9991006899, KMDF_PR_20200901_0234, NTIS, KMDF-RnD 202014X28-00]. The correct Funding statement appears below.

**FUNDING**

Korea Medical Device Development Fund grant funded by the Korean government [grant number: 9991006899, KMDF_PR_20200901_0234, NTIS, KMDF-RnD 202014X28-00 **(**RS-2020-KD000234)] The authors apologize for this error and state that this does not change the scientific conclusions of the article in any way. The original article has been updated.

